# Efficacy and safety of primary letermovir prophylaxis for cytomegalovirus infection in paediatric patients undergoing allogeneic transplantation: a single-centre, retrospective, real-world analysis

**DOI:** 10.46989/001c.131683

**Published:** 2025-03-14

**Authors:** Xin Wang, Chaoqian Jiang, Lipeng Liu, Xia Chen, Yuanyuan Ren, Yang Wan, Aoli Zhang, Xiaoyan Zhang, Yue Shang, Yao Zou, Xiaojuan Chen, Fang Liu, Wenyu Yang, Xiaofan Zhu, Ye Guo

**Affiliations:** 1 Department of Paediatric Hematology Institute of Hematology & Blood Diseases Hospital, Tianjin, China; 2 Tianjin Institutes of Health Science, Tianjin, China

**Keywords:** Hematopoietic Stem Cell Transplantation, Paediatrics, Cytomegalovirus, Infectious complications in hematological malignancies, Letermovir

## Abstract

**Background:**

Cytomegalovirus (CMV) infection is a common and life-threatening complication following allogeneic haematopoietic stem cell transplantation (allo-HSCT). Letermovir (LET) has been the standard prophylaxis for adult recipients, but studies in children remain limited.

**Methods:**

We retrospectively analyzed children with or without LET prophylaxis after haploidentical donor (HID) for the Beijing protocol or unrelated cord blood (UCB) transplantation.

**Results:**

Of the 151 patients, 67 received LET, including 35 HID recipients and 32 UCB recipients. During the 180 days after transplantation, we found that the LET group had a lower incidence of clinically significant CMV infection (csCMVi) than the non-LET group (13.4% vs. 56.0%, P＜0.001). In the LET group, later LET administration was identified as a risk factor for the occurrence of csCMVi (HR: 1.07, 95% CI: 1.01 - 1.14, P=0.029). Further, the HID subgroup had a lower incidence of csCMVi during follow-up than the UCB subgroup (2.9% vs. 25.0%, P=0.009). In terms of safety, the incidence and severity of adverse events, overall survival, cumulative incidence of relapse, relapse free survival, nonrelapse mortality and graft versus host disease-free, relapse-free survival were similar between the two groups.

**Conclusion:**

LET is effective and safe in preventing csCMVi among Chinese children undergoing allo-HSCT. Compared to UCB recipients, children undergoing HID transplantation for the Beijing protocol develop less scCMVi up to 180 days post-HSCT.

## Introduction

Cytomegalovirus (CMV) infection is a common and life-threatening complication following allogeneic hematopoietic stem cell transplantation (allo-HSCT). It is estimated that 10 to 40% of patients underwent HSCT will develop CMV infection, with a mortality rate as high as 70%. Notably, patients were defined as being a high risk of CMV disease if they received stem cells from unrelated cord blood (UCB) or haploidentical donors (HID), due to their delayed immune reconstitution (IR) and HLA disparity.^1,2^

Letermovir (LET) is a novel exclusive anti-CMV drug that acts by inhibiting the CMV terminase complex and has been successfully confirmed to reduce clinically significant CMV infection (csCMVi) with good tolerability. Since a landmark prospective, randomized, large-scale cohort studies,^3^ it has been recommended as the standard prophylaxis for adult seropositive recipients following allo-HSCT by the US Food and Drug Administration (FDA) and the European Conference on Infections in Leukaemia (ECIL 7).^1,3^ Subsequently, several studies also unequivocally demonstrated the efficacy and safety of LET in mitigating the risk of CMV disease, particularly among those identified as being at high risk.^4–6^ Although the partially published data from the ongoing Phase 2b non-randomized, multicenter, open-label, sequential dose escalation study (NCT03940586; MK-8228-030) indicateed the efficacy and safety of using adult doses of LET in children aged 12-18 years,^7^ the use of LET in pediatric patients was still considered off-label, especially in those under the age of 12. Currently, there are several small retrospective cohort studies that have confirmed the efficacy and safety of LET in children,^8–11^ but less studies have further explored it in the context of alternative donors.

Here, we retrospectively evaluated the efficacy and safety of primarily prophylactic LET for paediatric patients who underwent UCB or HID transplantation.

## Materials and Methods

### Patients and study design

A total of 151 children underwent allo-HSCT between January 2021 and December 2023 at the Institute of Hematology & Blood Diseases Hospital, Chinese Academy of Medical Sciences. Sixty-seven patients who received LET prophylaxis after allo-HSCT were compared retrospectively with eighty-four patients without.

Because infection affects 20% to 30% of CMV-seronegative recipients who have undergone transplantation from CMV-seropositive donors,^12^ and the effectiveness of LET in these recipients is specified in the Risk Management Plan mandated by the Pharmaceuticals and Medical Devices Agency (PMDA) of Japan,^13^ Our study included these recipients. The primary end point was the incidence of csCMVi within 180 days post-HSCT, which was defined as CMV viremia or the application of preemptive treatment (PET). The secondary end point was the occurrence of adverse events (AEs) and post-transplant outcomes. The median follow-up time for the LET group and the non-LET group was 417 (22-796) and 732.5 (96-1253) days, respectively.

### LET prophylaxis and CMV management

The dosages of letermovir were based on body surface area (BSA), specifically BSA/1.73×480mg. The patients who received concomitant cyclosporine (CsA) were administered half a dose of letermovir, BSA/1.73×240mg. According to the medication instructions, we began administering letermovir after allo-HSCT. The median interval from transplantation to LET administration was 10 (1-44) days, and the treatment was continued until day +100 post-transplantation.

We routinely measured the CMV-DNA load in plasma using real-time quantitative polymerase chain reaction (qPCR). This was done twice a week until neutrophil engraftment, then once a week until 100 days post-HSCT, and during subsequent outpatient follow-up visits. CMV DNAemia was defined in accordance with our institu tional guidelines as treatment initiated after CMV-DNA load >125 IU/mL (1000 copies/ml).^14^ Due to the bleeding tendency observed in the pediatric patients, the diagnosis of suspected CMV gastrointestinal disease in this study was based on clinical symptoms and CMV DNA in stool samples, without the conduct of histological biopsy. The anti-CMV drugs (intravenous ganciclovir or foscarnet) for PET were selected at the physician ’s discretion.

### Definitions

The hematopoietic cell transplantation-specific comorbidity index (HCT-CI) were defined according to Sorror et al.^15^ ADs included neutrophil graft failure, any organ impairment and orther infection. Neutrophil engraftment was defined as the first of 3 successive days with an absolute neutrophil count of ≥500/μl after post-transplantation.^16^ The hepatic, renal, and cardiac impairment was according to AST, ALT, Cr, and TnI levels, and their grade referring to the Common Terminology Criteria for Adverse Events (CTCAE - Version 5.0). The overall survival (OS), cumulative incidence of relapse (CIR), relapse free survival (RFS), nonrelapse mortality (NRM), graft versus host disease (GVHD), and GVHDfree, relapse-free survival (GRFS) were assessed as post-transplant outcomes and calculated from the date of HSCT. GRFS events were defined as grade III–IV acute GVHD (aGVHD), moderate to severe chronic GVHD (cGVHD), disease relapse, or death from any cause after HSCT.

### Statistical analyses

Continuous variables are expressed using the average (range), while categorical variables are represented using number (percentage). Categorical variables were appropriately evaluated using the Chi-square test, while continuous variables were assessed using the Mann-Whitney U test. A cumulative incidence curve was utilized for comparing cumulative incidence of csCMVi, GVHD, CIR and NRM. The OS, RFS, and GRFS were estimated using the Kaplan-Meier method and compared with the log-rank test. Cox proportional hazards model was conducted to assess the relationships between clinical factors and csCMVi. A P value greater than 0.05 was considered statistically insignificant when using a two-tailed test. Statistical analyses were performed using R version 4.3.0.

## Results

### Study Population

A total of 151 patients were included, consisting of 67 in the LET group and 84 in the non-LET group. The two groups had well balanced clinical and transplantation characteristics (Table 1). The preexisting disorders included lymphoma and bone marrow failure (BMF), acute myeloid leukemia (AML), acute lymphoblastic leukemia (ALL), myelodysplastic syndromes (MDS), myelodysplastic/myeloproliferative neoplamsms (MDS/MPN), hybrid acute leukemia (HAL). All HID recipients underwent the Beijing protocol, which included antithymocyte globulin (ATG), and none had received alemtuzumab or undergone ex vivo T-cell depletion

**Table 1.** Baseline characteristics of the all paediatric patients.

	**LET Group**	**Non-LET Group**	**P.value**
**n**	67	84	
**Sex**			1
Female	24 (35.8)	31 (36.9)	
Male	43 (64.2)	53 (63.1)	
**Age, years**			0.194
mean (range)	7.6 (1-17)	8.4 (1-19)	
**BMI**			0.837
mean (SD)	17.72 (3.75)	17.72 (3.99)	
**CMV antibody combination**			0.207
Donor+/Recipient+	27 (40.3)	44 (52.4)	
Donor+/Recipient-	14 (20.9)	10 (11.9)	
Donor-/Recipient+	26 (38.8)	30 (35.7)	
**Primary reason for transplantation**			1
Benign disease	18 (26.9)	23 (27.4)	
Malignant disease	49 (73.1)	61 (72.6)	
**HCT-CI**			0.132
0	54 (80.6)	75 (89.3)	
≥1	13 (19.4)	9 (10.7)	
**Stem cell source**			0.288
cord blood	32 (47.8)	33 (39.3)	
peripheral blood	25 (37.3)	30 (35.7)	
bone marrow+peripheral blood	10 (14.9)	21 (25.0)	
**Conditioning regimen**			1
MAC	49 (73.1)	62 (73.8)	
RIC/NMAC	18 (26.9)	22 (26.2)	
**Drugs used for GVHD prophylaxis**			0.967
Cyclosporin based	62 (92.5)	79 (94.0)	
Tacrolimus based	5 (7.5)	5 (6.0)	
**Type of donor**			0.379
UCB	32 (47.8)	33 (39.3)	
HID	35 (52.2)	51 (60.7)	
**ABO match graft**			0.716
Matched	33 (49.3)	45 (53.6)	
Mismatched	34 (50.7)	39 (46.4)	

^1^ LET, Letermovir; BMI, Body Mass Index; CMV, Cytomegalovirus; HCT-CI, Hematopoietic Cell Transplantation-specific Comorbidity Index; MAC, Myeloablative transplantation; RIC/NMAC, Reduced intensity conditioning/Non-MAC; GVHD, graft-versus-host disease; UCB, unrelated cord blood; HID, haploidentical donor; SD: standard deviation.

^2^ Values are reported as n (%) of patients, unless indicated otherwise.

^3^ P values were calculated using a two-sided Chi-Square Test for categorical variables and a Wilcoxon rank-sum test for continuous variables.

### Efficiency Analysis

**Figure 1.**
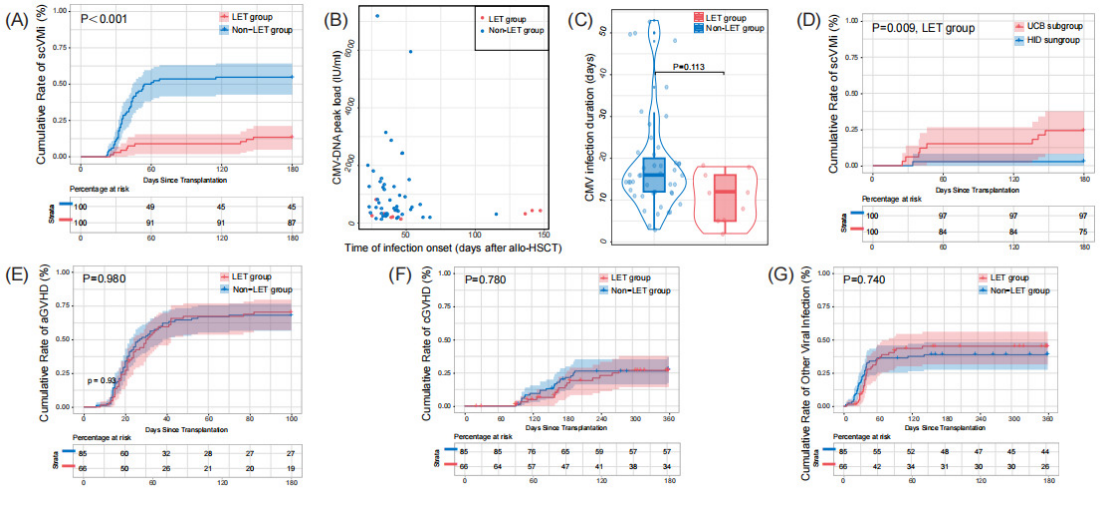
Panel A shows the cumulative incidence of clinically significant Cytomegalovirus infection (csCMVi), stratified by letermovir administration group, through 180 days after allogeneic hematopoietic cell transplantation (allo-HSCT). Panel B and C show the time of csCMVi onset, CMV-DNA peak load and csCMVi duration. Panel D shows the cumulative incidence of csCMVi through 180 days after allo-HSCT, stratified by donor type subgroup. Panel E, F and G show acute graft-versus-host disease (aGVHD), chronic graft-versus-host disease (cGVHD) and orther viral infection probability. All P values are two-sided.

**Figure 2.**
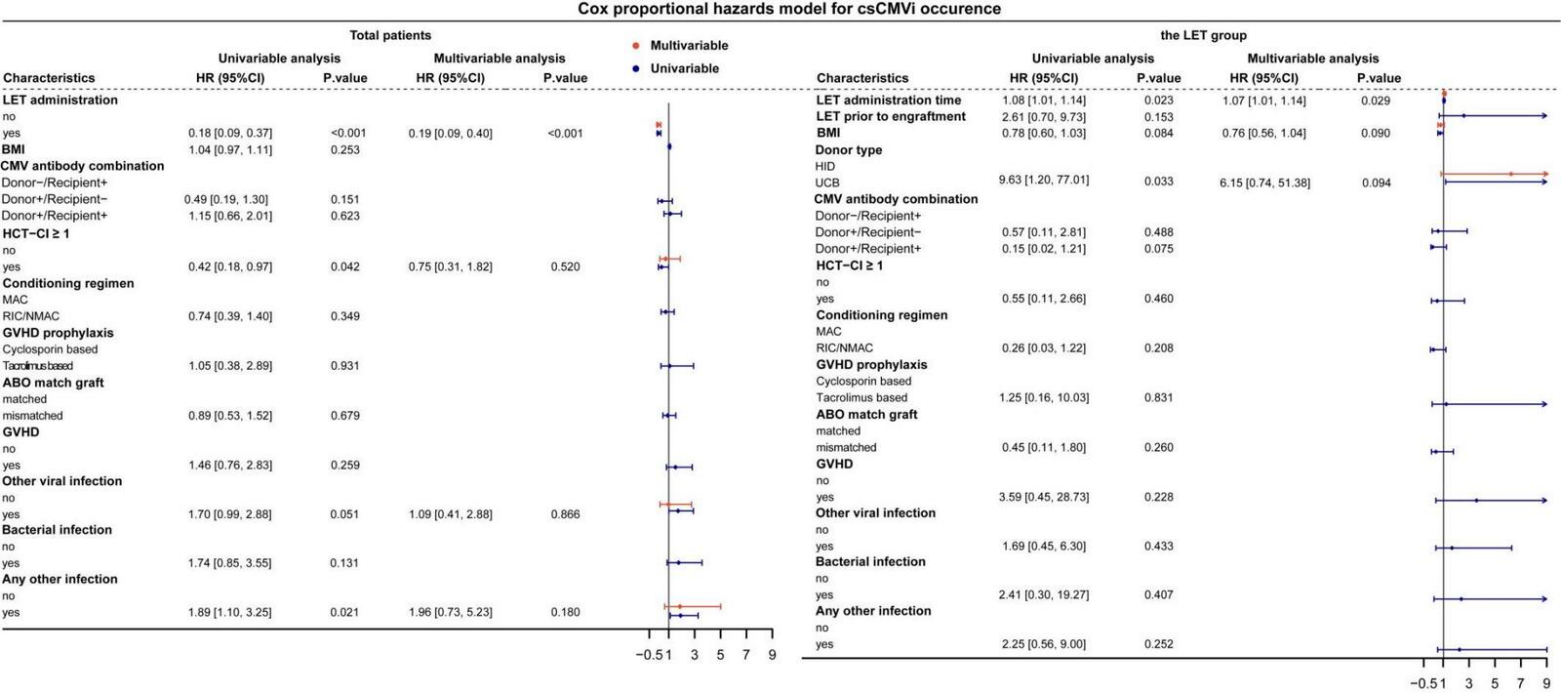
Forest plots showed the impact of clinical factors on the incidence of clinical significant Cytomegalovirus infection (csCMVi) in all patients and the letermovir group. Clinical factors included the administration of LET, body mass index (BMI), CMV serostatus of recipients, donor type, Hematopoietic Cell Transplantation-specific Comorbidity Index (HCT-CI) score more than one, the conditioning regimen, graft-versus-host disease (GVHD) prophylaxis, ABO match graft, the occurrence of GVHD prior to the csCMVi, and the occurrence of other types of viral, bacterial, or fungal infection prior to the csCMVi. Diamonds represent the hazard ratios, and the horizontal bars extend from the lower limit to the upper limit of the 95% confidence interval.

At the 180 days after transplantation, the cumulative incidence of csCMVi was significantly different between the LET group and the non-LET group (9/67 [13.4%] vs.46/84 [56.0%], P<0.001) (Figure 1A). Further, among patients who reached the primary end point, the LET group had later a later average time of infection onset (day +71 [25-147] vs.+41 [22-115] after transplantation, P=0.306), lower average CMV-DNA peak load (343 [136-811] vs. 1080 [134-7187] IU/ml, P=0.015) and shorter average duration (11 [2-18] vs. 19 [3-53] days, P=0.113) than the non-LET group (Figure 1B). In Cox proportional hazards model, LET administration was identified as an independent favourable factor for protecting children from csCMVi (hazard ratio [HR] 0.19, 95% CI 0.09 - 0.40, P<0.001) (Figure 2). A few patients developed suspected CMV infection, affecting the gastrointestinal tract in all cases, with 4/67 (6.0%) in the LET group and 6/84 (7.1%) in the non-LET group. Finally, all successfully treated with antiviral therapy (ganciclovir / foscarnet).

Among the LET group, there were 9 children developed csCMVi. Their clinical features prior to their first csCMVi were presented in Table 2. Their average age was 7.7 years, similar to the average age of the LET group, which was 7.6 years. All pediatric patients were administered Letermovir up to 100 days post-transplantation. Although a few patients experienced csCMVi after discontinuation of LET, they did not resume LET treatment. 6/67 (9.0%) developed breakthrough CMV infection, including 5 receiving UCB transplantation and 1 receiving HID transplantation (P=0.071). Their average time of csCMVi was day +36 (25 - 46) post-HSCT, their average CMV-DNA peak load was 319 (136 - 811) IU/ml, and their average duration of CMVi was 11 (5 - 18) days. After viral clearance, two patients (Pt 4 and Pt 6) developed recurrent csCMVi. Pt 4 developed breakthrough infection again at day +95 post-transplantation, while Pt 6 experienced reactivation at day +123 post-transplantation, which was also the 23 days after LET discontinuation. Besides them, after LET discontinuation, other three patients (Pt 7, Pt 8 and Pt 9) receiving UCB transplantation also developed csCMVi. The cumulative curve showed HID subgroup had significantly lower incidence of csCMVi than UCB subgroup (P=0.009, Figure 1D). The multivariable analysis suggested the late initiatial time of LET administration was risk for the occurence of csCMVi (HR 1.07, 95% CI 1.01 - 1.14, P=0.029) (Figure 2). All pediatric patients who developed GVHD were administered steroid-based immunosuppressive therapy, with gradual dose reduction upon symptomatic improvement. Among those who experienced GVHD, the incidence of csCMVi was also significantly lower in the LET group compared to the non-LET group (30/61, 49.2% versus 8/51, 15.7%, P < 0.001).

**Table 2.** Clinical manifestations before or at primary endpoint in patients within the LET group.

**ID**	**Pt 1**	**Pt 2**	**Pt 3**	**Pt 4**	**Pt 5**	**Pt 6**	**Pt 7**	**Pt 8**	**Pt 9**
**Sex**	M	M	F	F	F	M	F	F	M
**Age**	3	3	2	10	8	14	4	14	11
**CMV serostatus**	D-/R+	D-/R+	D+/R+	D-/R+	D-/R+	^*^D+/R-	D-/R+	^*^D+/R-	D-/R+
**Transplant-ation indication**	Leukemia	Leukemia	Leukemia	MDS	MDS/ MPN	Leukemia	MDS	Leukemia	Leukemia
**Conditioni-ng regimen**	MAC	MAC	MAC	MAC	MAC	MAC	MAC	MAC	MAC
**Letermovir start time**	8	44	23	30	21	2	9	32	2
**GVHD prophylaxis**	CsA based	CsA based	CsA based	CsA based	CsA based	CsA based	CsA based	CsA based	CsA based
**Donor type**	UCB	UCB	HID	UCB	UCB	UCB	UCB	UCB	UCB
**Neutrophil engraftme-nt**	yes	yes	yes	yes	yes	yes	yes	yes	yes
**aGVHD grade**	-	4	3	1	3	4	2	1	2
**cGVHD**	-	-	-	-	-	-	-	-	yes
**Other infection**	bacterial	-	-	-	fungal	BKV	-	-	BKV
**Primary end point**	day +25	day +28	day +34	day +39	day +40	day +46	day +136	day +141	day +147
**Peak CMV DNA load**	249 IU/ml	811 IU/ml	319 IU/ml	192 IU/ml	209 IU/ml	136 IU/ml	315 IU/ml	430 IU/ml	427 IU/ml
**Therapy**									
ganciclovir	yes	yes	yes	yes	yes	yes	yes	yes	yes
foscarnet	-	yes	-	-	yes	-	-	-	-

*CMV-IgG (+) in UCB indicates that the infant was exposed to CMV during the mother’s pregnancy, and the CMV IgG antibodies were transferred to the fetus through the mother.

Pt: patient; M, Male; F, Female; BMI, Body Mass Index; CMV, Cytomegalovirus; R: recipients; HCT-CI, Hematopoietic Cell Transplantation-specific Comorbidity Index; MAC, Myeloablative transplantation; GVHD, graft-versus-host disease; CsA: Cyclosporin A; UCB, unrelated cord blood; HID, haploidentical donor.

### Safety Analysis

There was no discontinuation due to toxicity was observed in our study. There was no engraftment failure observed in the LET group and the non-LET group, and the average time to neutrophil engraftment was 16 (10 - 34) days and 16 (9 - 28) days in patients with and without LET prophylaxis, respectively (P=0.490). For transplantation-related complication, although the LET group had a higher incidence of Epstein-Barr virus (EBV) infection compared to the non-LET group (9/67 [13.4%] vs.5/84 [6.0%], P=0.196), their subsequent incidence of post-transplantation lymphoproliferative disorder (PTLD) were similar (2/67 [3.0%] vs.2/84 [2.4%], P=1, Table 3). Besides, our data indicated that both groups exhibited comparable frequencies and severity of GVHD (Figure 1E), organ damage, and other infections (Figure 1G). The median aGVHD was 30 days and 27 days for the LET and non-LET groups, respectively (p = 0.061, Figure 1E). The median time to onset of aGVHD was 30 days and 27 days for the LET and non-LET groups, respectively (p = 0.061).

**Table 3.** Adverse Events.

**Adverse Event**	**LET group**	**Non-LET group**	**X-squared**	**P.value**	
	n=67	n=84			
**aGVHD**	47 (70.1)	60 (71.4)	0	1.000	
0 grade	20 (29.9)	27 (32.1)	3.418	0.332	
1 grade	11 (22.9)	16 (19.0)			
2 grade	9 (13.4)	18 (21.4)			
3-4 grade	27 (40.3)	23 (27.4)			
**cGVHD**	15 (22.4)	24 (28.6)	0.456	0.499	
mild	12 (17.9)	19 (22.6)	0.748	0.731	
moderate to severe	3 (4.5)	5 (6.0)			
**Hepatic impairment**	60 (89.6)	74 (88.1)	＜0.001	0.982	
0 grade	7 (10.4)	10 (11.9)	1.202	0.878	
1 grade	19 (28.4)	27 (32.1)			
2 grade	16 (23.9)	16 (19.0)			
3 grade	19 (28.4)	26 (31.0)			
4 grade	6 (9.0)	5 (6.0)			
**Renal impairment**	7 (10.4)	6 (7.1)	0.183	0.669	
0 grade	60 (89.6)	78 (92.9)	0.532	0.767	
1 grade	6 (9.0)	5 (6.0)			
2 grade	1 (1.5)	1 (1.2)			
**Cardiac impairment**	4 (6.0)	2 (2.4)	0.494	0.482	
0 grade	63 (94.0)	82 (97.6)	0.494	0.482	
1 grade	4 (6.0)	2 (2.4)			
**Any other infection**	32 (47.8)	40 (47.6)	0	1	
**Other viral infection**	29 (43.3)	33 (39.3)	0.109	0.742	
Epstein-Barr Virus infection	9 (13.4)	5 (6.0)	1.670	0.196	
PTLD	2 (3.0)	2 (2.4)	＜0.001	1	
Adenovirus infection	1 (1.5)	1 (1.2)	＜0.001	1	
BK Polyomavirus infection	20 (29.9)	27 (32.1)	0.016	0.900	
**Bacterial infection**	4 (6.0)	13 (15.5)	2.487	0.115	
**Fungal infection**	3 (4.5)	4 (4.8)	＜0.001	1	
**OS**	60 (89.6)	80 (95.2)	2.001	0.220	
*CIR	8 (16.7)	5 (8.1)	0.985	0.321	
*RFS	56 (83.6)	76 (90.5)	1.262	0.261	
*NRM	3 (6.3)	2 (3.2)	0.570	0.648	
*GRFS	40 (59.7)	61 (72.6)	3.268	0.071	

*Subjects: Malignant disease children，LET group: n=48，non-LET group: n=62.

aGVHD: Acute Graft-versus-Host Disease; cGVHD: Chronic Graft-versus-Host Disease; PTLD: Post Transplant Lymphoproliferative Disease; OS, overall survival; CIR, cumulative incidence of relapse; RFS, relapse free survival; NRM, nonrelapse mortality; GRFS, GVHD-free, relapse-free survival.

The main post-HSCT outcomes are summarized in Table 3. The causes of death among the 11 children included relapse, non-CMV severe infection and transplantation-associated thrombotic microangiopathy (TA-TMA). In each of the LET and non-LET groups, the 1-year OS of 89.6% and 95.2% (p = 0.220), CIR of 16.7% and 8.1% (p = 0.321), RFS of 83.6% and 90.5% (p = 0.261), NRM of 6.3% and 3.2% (p = 0.648) and GRFS of 59.7% and 72.6% (p = 0.071), respectively, were observed (Table 3). There were also no statistically significant differences in OS, CIR, RFS, NRM, and GRFS between the two groups in the cumulative incidence curves. The median GRFS for the LET group was 175 days, the median GRFS for the non-LET group was not reached.

**Figure 3.**
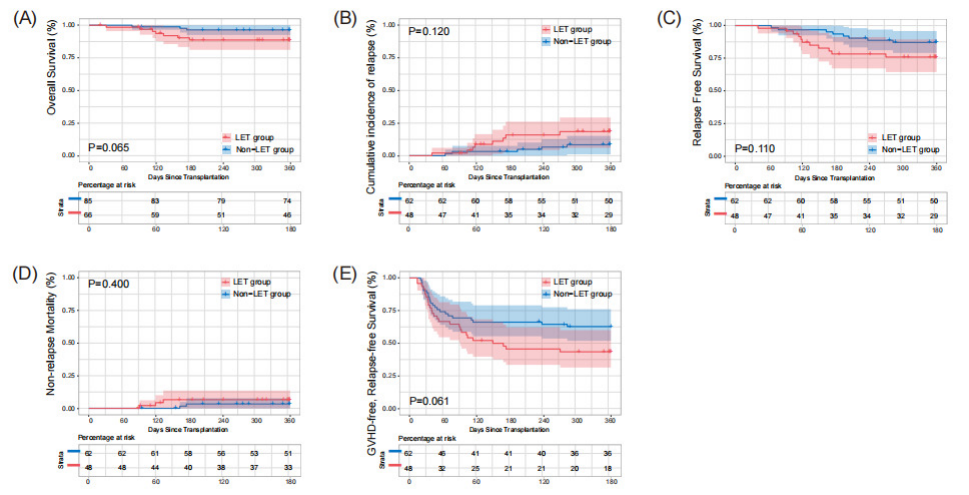
Panel A shows the overall survival (OS) for all children. Panel B, C, D and E show the cumulative incidence of relapse (CIR), relapse free survival (RFS), nonrelapse mortality (NRM) and graft-versus-host disease-free, relapse-free survival (GRFS) in children with malignant diseases. All P values are two-sided.

## Discussion

The study retrospectively analyzed the efficacy and safety of LET prophylaxis in children undergoing HID and UCB transplantation. In addition, we focused on its efficacy in paediatric patients who received transplants from different donor sources.

Among 151 paediatric patients who underwent alternative donor transplantation in our center both before and after the the introduction of LET in China (August 2022), we found that LET was highly effective in preventing CMV infection. Despite the fact that all our study populations were considered high-risk for CMV infection, the reported rates of breakthrough infections (8/39, 20.5%) and the incidence of endpoint events within 180 days post-transplantation (4/41, 9.8%) from previous paediatric studies were comparable to our findings.^8,9^ It is noteworthy that both our and previous paediatric studies have reported lower infection rates compared to the phase 3 clinical trial,^3^ suggesting that the age of the recipients may be a major factor contributing to this discrepancy. Raheel Iftikhar et al. found that being older than 12 years was an independent risk factor for CMV infection, through analysis of 230 consecutive patients undergoing allo-HSCT.^17^ In addition, age-related thymic insufficiency also contributes to differences in CD4+ and CD8+ T cells reconstitution patterns between children and adults after transplantation.^18^ Besides, the phase 3 clinical trial enrolled only R+ patients, whereas approximately 20% of our study recipients had a negative CMV serostatus, which may partly contribute to the observed difference. Overall, our study highlights the significant efficacy of LET in preventing CMV infection, even in high-risk paediatric patients.

We then conducted a further analysis of the paediatric patients in the LET group. Our study first compared the efficacy of LET in paediatric patients undergoing HID for the Beijing protocol and UCB transplantation. We found that the efficacy of LET in HID recipients for the Beijing protocol exceeded that in UCB recipients. During prophylaxis, 5 out of 6 patients who developed a breakthrough infection received a UCB transplantation. After discontinuation of LET, all 3 cases of late-onset CMV infection were from the UCB subgroup. This may be related to the lower total lymphocyte count in UCB grafts, leading to delayed IR post-transplantation.^19^ A retrospective study by Hill et al. of 61 patients who received a UCB transplantation, of whom 21 received LET prophylaxis, also suggested this. After one year of follow-up, they found that the cumulative incidence of CMV infection was almost similar between the group receiving LET prophylaxis and the group not receiving LET prophylaxis.^20^ Therefore, long-term monitoring of children undergoing UCB transplantation after discontinuation of LET was indeed necessary. To further optimize the prophylactic strategy, extensive long-term research is essential to investigate the feasibility of extending the duration of LET use in UCB recipients. Most importantly, our results showed that early use of LET after transplantation significantly reduced the risk of CMV infection. This important finding, which has not yet been reported in children, requires further validation and consolidation through larger cohort studies. Such efforts will also help determine the optimal timing of LET administration.

In terms of safety, previous pediatric studies have predominantly determined the dosage of letermovir based on BSA and concurrent CsA use, with all of them validating its favorable efficacy and safety, and our study also adhered to this principle. Nevertheless, age continued to be a crucial factor that cannot be overlooked in pediatric medication. An ongoing Phase 2 clinical trial has divided patients into three age groups to explore optimal dosing strategies.^7^ Published data from this trial has indicated that children aged 12-18 could safely receive the same LET dosage as adults, which was consistent with the dosing administered to this age group in our study.

During follow-up, we did not observe any LET-related myelosuppression, organ toxicity or transplant-related complications. The absence of myelosuppression with LET was a prerequisite for its use prior to neutrophil engraftment, thereby ensuring its safety. In line with similar findings in previous studies, the study confirmed that the time to neutrophil engraftment was comparable between patients who received LET and those who did not (P=0.953).^21^ Notably, although the EBV infection rate was slightly higher in the LET group compared to the non-LET group, this difference was not statistically significant. In addition, the incidence of PTLD was similar between the two groups. Several recent studies have suggested that LET may increase the risk of EBV infection and PTLD in adults,^22,23^ but there are few reports in children.

This study was a small-sample, single-center, retrospective real-world study. Additionally, there was no established dosing protocol for LET in children, resulting in a considerable time span for the initiation of LET in the LET group. Furthermore, although most of the patients included in the non-LET group underwent transplantation before the approval of LET (August 2022), a portion of them received allo-HSCT recently without LET prophylaxis. Due to the advent of new antibiotics, diagnostic techniques and so on,^24^ the clinical outcomes of patients undergoing transplantation recently have improved. This may also impact the results of this study.

In conclusion, LET demonstrates efficacy in preventing csCMVi among Chinese children undergoing alternative donor transplantation, especially in HID recipients, and also has a good safety profile.

### Authors’ Contribution

Conceptualization: Ye Guo, Xiaofan Zhu; Data curation: Xia Chen, Yuanyuan Ren, Yang Wan, Aoli Zhang, Xiaoyan Zhang, Yue Shang, Yao Zou, Xiaojuan Chen, Fang Liu, Wenyu Yang; Formal Analysis: Xin Wang, Chaoqian Jiang, Lipeng Liu; Writing – original draft: Xin Wang, Chaoqian Jiang, Lipeng Liu; Writing – review & editing: Ye Guo, Xiaofan Zhu.

### Competing of Interest – COPE

The authors have no conflicts of interest declared.

### Ethical Conduct Approval – Helsinki – IACUC

The retrospective studie design and methods complied with the Declaration of Helsinki and were approved by the Ethics Committee and Institutional Review Board of the Institute of Hematology and Blood Diseases Hospital, Chinese Academy of Medical Sciences & Peking Union Medical College.

### Informed Consent Statement

All authors and institutions have confirmed this manuscript for publication.

### Data Availability Statement

All are available upon reasonable request.

## References

[ref-420568] Ljungman P.. (2019). Guidelines for the management of cytomegalovirus infection in patients with haematological malignancies and after stem cell transplantation from the 2017 European Conference on Infections in Leukaemia (ECIL 7). The Lancet Infectious Diseases.

[ref-420569] Velardi E.. (2021). The role of the thymus in allogeneic bone marrow transplantation and the recovery of the peripheral T-cell compartment. Semin Immunopathol.

[ref-420570] Marty F. M., Ljungman P. (2017). Letermovir Prophylaxis for Cytomegalovirus in Hematopoietic-Cell Transplantation. The new england journal of medicine.

[ref-420571] Nakagawa Daishi, Shimomura Yoshimitsu, Mitsuyuki Satoshi (2023). Efficacy of letermovir in HLA-haploidentical hematopoietic transplantation with posttransplant cyclophosphamide. International Journal of Hematology.

[ref-420572] Toya Takashi, Mizuno Kota, Sakurai Masatoshi (2023). Differential clinical impact of letermovir prophylaxis according to graft sources: a KSGCT multicenter retrospective analysis. Blood Advances.

[ref-420573] Yan B.. Letermovir prophylaxis reduced cytomegalovirus reactivation and resistance post umbilical cord blood transplantation.

[ref-420574] Groll A. H.. (2024). Pharmacokinetics, Safety, and Efficacy of Letermovir for Cytomegalovirus Prophylaxis in Adolescent Hematopoietic Cell Transplantation Recipients. Pediatric Infectious Disease Journal.

[ref-420575] Chen T. T., David A. P., Barthelmess E. K., MacBrayne C. E. (2023). Letermovir for Cytomegalovirus prophylaxis in pediatric hematopoietic stem cell transplantation. Pediatric Blood & Cancer.

[ref-420576] Galaverna Federica, Baccelli Francesco, Zama Daniele (2024). Letermovir for Cytomegalovirus infection in pediatric patients undergoing allogenic hematopoietic stem cell transplantation: a real-life study by the Infectious Diseases Working Group of Italian Association of Pediatric Hematology-Oncology (AIEOP). Bone Marrow Transplantation.

[ref-420577] Pfeiffer T.. (2024). Letermovir as cytomegalovirus prophylaxis in children undergoing allogeneic hematopoietic cell transplantation. Bone Marrow Transplant.

[ref-420578] César T.. (2024). Letermovir for CMV Prophylaxis in Very High-Risk Pediatric Hematopoietic Stem Cell Transplantation Recipients for Inborn Errors of Immunity. J Clin Immunol.

[ref-420579] Einsele H., Ljungman P., Boeckh M. (2020). How I treat CMV reactivation after allogeneic hematopoietic stem cell transplantation. Blood.

[ref-420580] Hiraishi I., Ueno R., Watanabe A., Maekawa S. (2021). Safety and Effectiveness of Letermovir in Allogenic Hematopoietic Stem Cell Transplantation Recipients: Interim Report of Post-marketing Surveillance in Japan. Clin Drug Investig.

[ref-420581] Ljungman P.. (2017). Definitions of Cytomegalovirus Infection and Disease in Transplant Patients for Use in Clinical Trials: Table 1. Clin Infect Dis.

[ref-420582] Sorror M. L.. (2005). Hematopoietic cell transplantation (HCT)-specific comorbidity index: a new tool for risk assessment before allogeneic HCT. Blood.

[ref-420583] Sureda A.. (2024). Harmonizing definitions for hematopoietic recovery, graft rejection, graft failure, poor graft function, and donor chimerism in allogeneic hematopoietic cell transplantation: a report on behalf of the EBMT, ASTCT, CIBMTR, and APBMT. Bone Marrow Transplant.

[ref-420584] Iftikhar R.. (2023). Cytomegalovirus Infection Post-Allogeneic Stem Cell Transplantation: Experience from a Country with High Seropositivity. Transplantation and Cellular Therapy.

[ref-420585] Kalwak K.. (2002). Immune reconstitution after haematopoietic cell transplantation in children: immunophenotype analysis with regard to factors affecting the speed of recovery. Br J Haematol.

[ref-420586] Rénard C.. (2011). Lymphocyte subset reconstitution after unrelated cord blood or bone marrow transplantation in children. Br J Haematol.

[ref-420587] Hill Joshua A., Zamora Danniel, Xie Hu (2021). Delayed-onset cytomegalovirus infection is frequent after discontinuing letermovir in cord blood transplant recipients. Blood Advances.

[ref-420588] Yoshimura H.. (2022). Real-world efficacy of letermovir prophylaxis for cytomegalovirus infection after allogeneic hematopoietic stem cell transplantation: A single-center retrospective analysis. Journal of Infection and Chemotherapy.

[ref-420589] Zhen Sisi. (2024). Infectious Complications (Glasgow).

[ref-420590] Pei Xuying, Huang Xiaojun (2024). Infectious Complications (Glasgow).

[ref-420591] Gooley T. A., Sorror M. L., Marr K. A. (2010). Reduced Mortality after Allogeneic Hematopoietic-Cell Transplantation. n engl j med.

